# Platelet integrin αIIbβ3: signal transduction, regulation, and its therapeutic targeting

**DOI:** 10.1186/s13045-019-0709-6

**Published:** 2019-03-07

**Authors:** Jiansong Huang, Xia Li, Xiaofeng Shi, Mark Zhu, Jinghan Wang, Shujuan Huang, Xin Huang, Huafeng Wang, Ling Li, Huan Deng, Yulan Zhou, Jianhua Mao, Zhangbiao Long, Zhixin Ma, Wenle Ye, Jiajia Pan, Xiaodong Xi, Jie Jin

**Affiliations:** 1 Department of Hematology, The First Affiliated Hospital, Zhejiang University School of Medicine, Hangzhou, Zhejiang China; 2 Key Laboratory of Hematologic Malignancies, Diagnosis and Treatment, Hangzhou, Zhejiang China; 3 Institute of Hematology, Zhejiang University School of Medicine, Hangzhou, Zhejiang China; 4 Department of Hematology, Affiliated Hospital of Jiangsu University, Zhenjiang, Jiangsu China; 5 Department of Hematological Malignancies Translational Science, Gehr Family Center for Leukemia Research, Hematologic Malignancies and Stem Cell Transplantation Institute, Beckman Research Institute, City of Hope Medical Center, Duarte, CA 91010 USA; 6 Department of Pathology, The Fourth Affiliated Hospital of Nanchang University, Nanchang, Jiangxi China; 7 Department of Hematology, The First Affiliated Hospital of Nanchang University, Nanchang, Jiangxi China; 8 State Key Laboratory of Medical Genomics, Shanghai Institute of Hematology, Collaborative Innovation Center of Hematology, Ruijin Hospital Affiliated to Shanghai Jiao Tong University School of Medicine, Shanghai, China; 9 Sino-French Research Centre for Life Sciences and Genomics, Ruijin Hospital Affiliated to Shanghai Jiao Tong University School of Medicine, Shanghai, China; 10 Department of Hematology, The First Affiliated Hospital of Anhui Medical University, Hefei, China; 11 Clinical Prenatal Diagnosis Center, Women’s Hospital, Zhejiang University School of Medicine, Hangzhou, Zhejiang China

**Keywords:** Integrin αIIbβ3, Signal transduction, Talin, Kindlin, Transmembrane proteins, Therapeutic targeting

## Abstract

Integrins are a family of transmembrane glycoprotein signaling receptors that can transmit bioinformation bidirectionally across the plasma membrane. Integrin αIIbβ3 is expressed at a high level in platelets and their progenitors, where it plays a central role in platelet functions, hemostasis, and arterial thrombosis. Integrin αIIbβ3 also participates in cancer progression, such as tumor cell proliferation and metastasis. In resting platelets, integrin αIIbβ3 adopts an inactive conformation. Upon agonist stimulation, the transduction of inside-out signals leads integrin αIIbβ3 to switch from a low- to high-affinity state for fibrinogen and other ligands. Ligand binding causes integrin clustering and subsequently promotes outside-in signaling, which initiates and amplifies a range of cellular events to drive essential platelet functions such as spreading, aggregation, clot retraction, and thrombus consolidation. Regulation of the bidirectional signaling of integrin αIIbβ3 requires the involvement of numerous interacting proteins, which associate with the cytoplasmic tails of αIIbβ3 in particular. Integrin αIIbβ3 and its signaling pathways are considered promising targets for antithrombotic therapy. This review describes the bidirectional signal transduction of integrin αIIbβ3 in platelets, as well as the proteins responsible for its regulation and therapeutic agents that target integrin αIIbβ3 and its signaling pathways.

## Background

Integrins, a family of transmembrane glycoprotein signaling receptors, comprise two distinct, noncovalently associated subunits, α and β. Each subunit consists of a large extracellular domain that contributes to ligand binding, a single-pass transmembrane (TM) domain, and a smaller unstructured cytoplasmic tail of approximately 20~70 amino acids (except β4). The cytoplasmic tail provides binding sites for adaptors, signaling proteins, and cytoskeleton-associated proteins, which play an essential role in integrin bidirectional signaling (inside-out signaling and outside-in signaling) [1]. In mammals, 18 α and 8 β subunits can combine in a restricted manner to form at least 24 integrins, which exhibit considerably different ligand binding properties, resulting in wide-ranging impacts on cellular functions, such as cell adhesion, spreading, migration, survival, differentiation, proliferation, and apoptosis [1]. Integrins can be grouped into 8 subgroups based on the identity of their β subunits (β1, β2, β3, β4, β5, β6, β7, and β8) [1]. Two subgroups of integrins are present in human platelets: β1 and β3, which account for a total of five human platelet integrins. Three β1 integrins are found in platelets, namely, α2β1, α5β1, and α6β1, which support platelet adhesion to the extracellular matrix proteins collagen [2], fibronectin [3], and laminin [4, 5], respectively. Two β3 integrins are present on platelets, namely, αvβ3 and αIIbβ3 [4, 5]. A variety of cells, including endothelial cells, smooth muscle cells, and fibroblasts, express αvβ3. However, there are only a few hundred copies of integrin αvβ3 per platelet [6, 7], and its function in platelets remains poorly understood. By contrast, αIIbβ3, also known as the glycoprotein GPIIb/IIIa (CD41/CD61) complex, is the dominant integrin on platelets and is essential for normal platelet functions. Integrin αIIbβ3 was also found to be expressed in tumor cells [8]. Integrin αIIbβ3 can bind to several arginine-glycine-aspartic acid (RGD)-containing ligands, including fibrinogen, fibrin, von Willebrand factor (vWF), and fibronectin. Of these ligands, fibrinogen is the major ligand. Integrin αIIbβ3 also interacts with the KQAGDV sequence of the fibrinogen γ-chain to cross-link platelets [9]. Glanzmann’s thrombasthenia (GT) is a rare autosomal recessive bleeding disorder that arises from disrupted αIIb and/or β3 subunit synthesis and function due to missense, nonsense, frame shift, or point mutations and exon skipping in the αIIb or β3 genes. This disruption impairs normal platelet functions, such as adhesion, spreading, and aggregation [10–12]. However, nonphysiological αIIbβ3-mediated platelet activation and aggregation often cause pathological arterial thrombosis [13].

Quantitative studies using 7E3 mAbs eventually confirmed that each unstimulated platelet presents approximately 50,000–100,000 copies of αIIbβ3 on its surface [14], and additional αIIbβ3 molecules in the α-granule membranes are recruited to the platelet surface during platelet secretion, particularly by stimulatory agonists, such as thrombin or adenosine diphosphate (ADP) [15, 16]. A critical characteristic of αIIbβ3 is that it can transmit bidirectional signaling. In resting platelets, integrin αIIbβ3 adopts an inactive conformation. In this state, the extracellular domain has low affinity for its ligands. However, upon agonist stimulation, the cytoplasmic tails of integrin αIIbβ3 are bound by intracellular proteins, particularly talin and/or kindlin. Binding triggers an unclasping of the intracellular and transmembrane αIIbβ3 complex, leading to a conformational change in the extracellular domain. This conformational change leads αIIbβ3 to switch from low affinity (inactive) to high affinity (active) for its ligand (fibrinogen). This process is known as inside-out signaling or integrin αIIbβ3 activation. The outside-in signaling of αIIbβ3 on platelets is triggered by the binding of fibrinogen to activated integrin αIIbβ3, leading to a cascade of intracellular signaling events that mediate irreversible stable adhesion, spreading, clot retraction, irreversible aggregation, and cytoskeletal reorganization of platelets, as well as subsequent thrombus growth.

Bidirectional signaling of integrin αIIbβ3 is vital for platelet functions, hemostasis, and arterial thrombosis. Bidirectional signaling of integrin αIIbβ3 also plays an important role in cancer progression through regulating the interaction of integrin αIIbβ3 with the fibrinogen/αvβ3 complex on the surface of tumor cells [8] and/or releasing vascular endothelial growth factor (VEGF) from activated platelets [17]. An improved understanding of integrin αIIbβ3 signal transduction and regulation will result in greater progress in understanding thrombosis and developing therapeutic agents. Several excellent reviews have provided an overview of the structure of integrin αIIbβ3 and its bidirectional signaling [18–23]. This brief review describes platelet integrin αIIbβ3 bidirectional signaling, the proteins responsible for regulating signal transduction, and the therapeutic agents targeting integrin αIIbβ3 and/or its signaling.

## Integrin αIIbβ3 inside-out signaling

The inside-out signaling of αIIbβ3 on platelets can be initiated by various soluble agonists, such as epinephrine, ADP, thromboxane A2 (TXA2), or thrombin, which bind to G protein-coupled seven-transmembrane domain receptors (GPCRs). Inside-out signaling can also be initiated by immobilized agonists, such as vWF or collagen, which mainly interact with GPIb-IX-V or GPVI, respectively. Inside-out signaling includes (1) intracellular activators (such as talin or kindlin) binding to integrin αIIbβ3 tails, (2) separation of the α and β TM and the cytoplasmic tail, (3) a conformational change of the extracellular domain of αIIbβ3, and (4) increasing ligand binding affinity and avidity. To date, talin, kindlin, and other proteins have been identified as directly or indirectly interacting with integrin cytoplasmic tails to participate in the inside-out signaling of αIIbβ3 [24].

### Talin

**Fig. 1:**
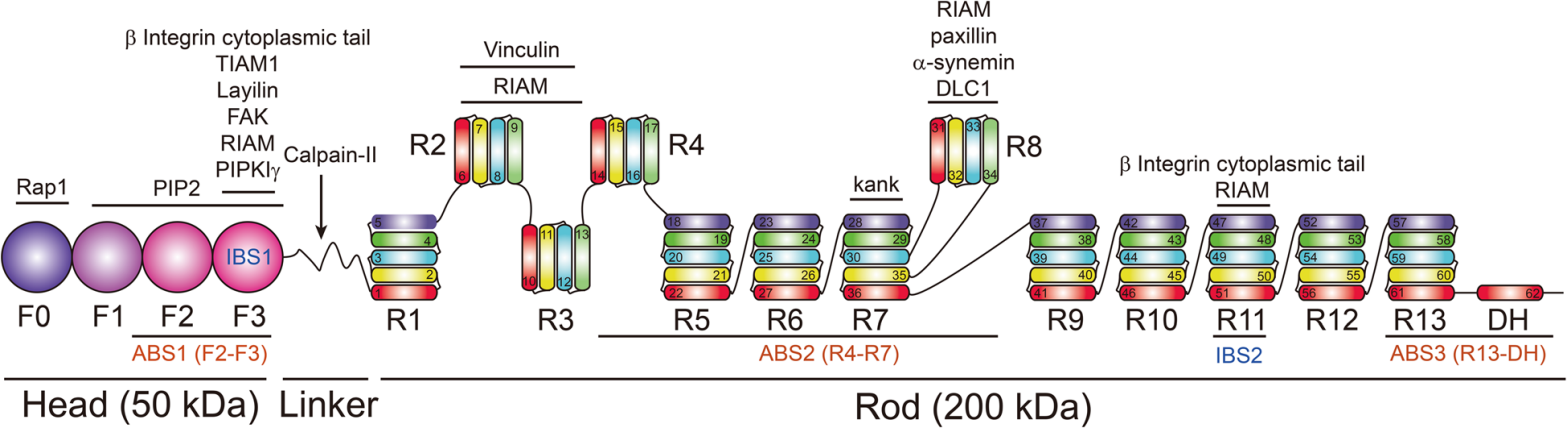
Domains and binding sites of talin. Talin-H comprises an atypical FERM domain containing F0, F1, F2, and F3 domains. Talin-R is composed of 13 amphipathic helical bundle domains (R1-R13, containing 62 α-helices), each consisting of four or five α-helices. A calpain-II cleavage site lies between talin-H and talin-R. Binding sites for interacting proteins are indicated by horizontal lines

Talin has long been known to play an essential role in integrin activation. As an integrin-actin adaptor protein, it is an autoinhibited dimer with a head-to-tail conformation [25]. It consists of a globular N-terminal head (talin-H, approximately 50 kDa) and a large flexible C-terminal rod region (talin-R, approximately 200 kDa) (Fig. 1) [26]. There is a short linker sequence containing a calpain-II cleavage site between the talin-H and talin-R regions [27]. The talin-H region contains an F0 subdomain and a so-called 4.1, ezrin, radixin, moesin (FERM) domain, comprising three subdomains named F1, F2, and F3. The F3 subdomain has a phosphotyrosine-binding domain (PTB)-like fold [28], which binds with high affinity to the first (or membrane-proximal) of two conserved NPXY motifs in the β tails at integrin-binding site 1 (IBS1) [29]. The F3 subdomain can also interact with phosphatidylinositol 4-phosphate 5-kinase isoform 1γ (PIPK1γ) [30], layilin [31], and focal adhesion kinase (FAK) [32]. The talin-R region is composed of 13 amphipathic helical bundle domains (R1-R13, containing 62 α-helices), each consisting of four or five α-helices. The talin-R region contains at least two actin-binding sites [33], a second integrin-binding site (IBS2) [34], and multiple binding sites for vinculin [35]. Thus, talin-H binds to the evolutionarily conserved NPXY motif of the β cytoplasmic tails of integrins, connecting the integrin with the actin cytoskeleton through the actin-binding site of talin-R.

Over the past 20 years, studies in cultured cells [36], mouse models [37, 38], and purified protein-reconstituted systems [39] have reinforced the notion that talin is an essential regulator of integrin ligand affinity. Binding of talin-H to the conserved N^744^PLY^747^ motif of the β3 tail is proposed to induce αIIbβ3 activation by disrupting the salt bridge between αIIb and the β3 tail [21]. Talin-H is sufficient to induce integrin activation, as evidenced by the fact that talin-H was able to induce integrin αIIbβ3 binding to the activation-specific mAb PAC-1 [40]. Studies on murine embryonic stem cell-derived megakaryocytes with talin knockdown have shown that talin is required for integrin αIIbβ3 activation in response to different agonists [41]. Furthermore, mice expressing the L^746^A mutation of β3 integrin, which is believed to selectively disrupt the interaction between αIIbβ3 and talin, display impaired inside-out activation of αIIbβ3 [42]. Conditional deletion of *talin-1* in mice showed that integrin αIIbβ3 is unable to activate in response to any tested agonists [43, 44]. This finding suggests that talin plays a crucial role in homeostasis and that talin is required for the activation and function of αIIbβ3 in vivo [43, 44]. Thus, disruption of the interaction of talin with integrin β3 may offer a strategy for anti-thrombosis [42, 45]. Recent data utilizing phospholipid nanodiscs bearing a single lipid-embedded integrin have also shown that talin-H binding to the integrin β3 tail is sufficient for integrin activation in the absence of other proteins [39]. However, solid evidence has clearly demonstrated that integrin activation also requires the cooperation of kindlin alongside talin [46–51].

### Kindlin

A series of publications have established a requirement for kindlin coordinating with talin for integrin αIIbβ3 inside-out signaling [47, 48, 52, 53]. In mammals, there are three evolutionarily conserved members of the kindlin family: kindlin-1, kindlin-2, and kindlin-3 [54, 55]. Kindlin-1 is ubiquitously expressed in epithelial cells, and kindlin-2 is broadly expressed in all solid tissues of mesenchymal origin. In contrast, kindlin-3 is mainly restricted to hematopoietic cells [56, 57]. However, recent experimental work has shown that kindlin-3 is also expressed in endothelial cells [58]. Mutations in the *kindlin-1* gene lead to Kindler syndrome, which is characterized by serious skin blistering, progressive poikiloderma, photosensitivity, and atrophy of the skin [59, 60]. Mutations in the *kindlin-3* gene lead to type-III leukocyte adhesion deficiency (LAD-III), as well as recurrent infections, immune deficiencies, and severe bleeding disorders caused by the dysfunction of integrins in leukocytes and platelets; loss of hematopoietic stem cells (HSCs) and hematopoietic progenitor cells (HPCs) in the bone marrow; elevated leukocyte counts; and osteopetrosis [61–64]. To date, no human diseases have been associated with mutations of the *kindlin-2* gene, but kindlin-2 is highly expressed in cancers of the lung, prostate, pancreas, liver, and esophagus [65]. Knockout of *kindlin-2* is embryonically lethal in mice and causes multiple severe abnormalities in zebrafish due to impaired integrin activation [49, 66, 67].

Using Chinese hamster ovary (CHO) cells expressing integrin αIIbβ3, the Calderwood group reported that kindlin-1 and talin cooperatively enhance integrin αIIbβ3 activation [52, 68] and that kindlin-2 is also a coactivator of talin-H in regulating integrin αIIbβ3 activation [48, 49]. Using *kindlin-3*^*−/−*^ mice, Moser et al. showed that in platelets lacking kindlin-3, integrin αIIbβ3 could not be activated despite normal talin expression [47]. Kindlin itself is incapable of unclasping the intracellular and transmembrane αIIbβ3 complex [69], and consequently, it is insufficient to trigger effective inside-out signaling of integrin αIIbβ3 [48]. However, there is a lack of evidence for the direct interaction between kindlins and talin-H [69]. Further studies will be required to address the unanswered question of how kindlin cooperates with talin to induce integrin activation. The tyrosine phosphorylation of the membrane-proximal N^744^PLY^747^ motif of the integrin β3 tail negatively regulates talin binding [70, 71]. Similar to talin, tyrosine phosphorylation of the membrane-distal N^756^ITY^759^ motif also inhibits kindlin-2 binding [46]. These observations suggest that transitions between the phosphorylated and nonphosphorylated states of the integrin β3 tail affect talin/kindlin-regulated integrin activation [46]. Tyrosine phosphorylation of the β3 tail also regulates β3 cleavage by calpain [72]. Structures of the kindlin-2/β-tail complex showed that the dimeric forms of kindlin-2 can bridge talin-activated integrins and promote integrin clustering [73]. Recent studies revealed that integrin-linked kinase (ILK) can interact with the F2 subdomain of kindlin-2 with high affinity and support αIIbβ3 activation [74, 75]. ADAP, a hematopoietic-specific adapter protein, is physically proximal to talin and kindlin-3 in human platelets. ADAP, when acting as a bridging molecule between kindlin and talin, promotes platelet integrin αIIbβ3 activation [38, 76, 77]. The paxillin (PXN) family members (paxillin and Hic-5) act as bridging molecules and are also able to promote platelet integrin αIIbβ3 activation by cooperating with kindlin and talin [51, 78, 79]. However, the exact details of how ILK, ADAP, paxillin, and Hic-5 assist kindlin and talin in mediating αIIbβ3 activation remain largely unknown.

### Other proteins that positively regulate integrin αIIbβ3 activation

In addition to talin and kindlin, other proteins, such as ILK [80], β3-endonexin [81, 82], calcium- and integrin-binding protein 1 (CIB1) [83, 84], chloride channel regulatory protein (ICln) [85], catalytic subunit of protein phosphatase 1 γ (PP1cγ) [86], and vinculin [87], may be involved in integrin αIIbβ3 activation. However, little is known about how these proteins exert effects on integrin activation and signaling. In addition to interacting with kindlin, ILK serves as an adaptor protein that forms the ILK/PINCH/parvin (IPP) complex with PINCH and parvins. The IPP complex interacts directly with the β3 cytoplasmic tail via ILK and regulates integrin activation in platelets. Loss of ILK has been reported to inhibit integrin activation, as assessed by the binding of soluble fibrinogen and PAC-1 [75, 80, 88, 89]. Platelets stimulated by ADP or phorbol 12-myristate 13-acetate (PMA) exhibited an increase in ILK activity associated with phosphorylation of β3 [90]. *ILK*^*−/−*^mice showed increased bleeding time, reduced aggregation, soluble fibrinogen binding, and defects in α-granule secretion [88]. These observations suggested that ILK may be involved in integrin αIIbβ3 inside-out and outside-in signaling. β3-Endonexin is a molecule that is known to induce αIIbβ3 activation in CHO cells by interacting with the N^756^ITY^759^ motif of the integrin β3 cytoplasmic tail. β3-Endonexin is present in resting human platelets. Nonetheless, there is little available information about how β3-endonexin regulates integrin αIIbβ3 [91, 92]. CIB1 can disrupt the association of αIIb and β3 by binding to the αIIb cytoplasmic tail, which in turn activates integrin αIIbβ3 [83, 93]. However, CIB1 has also been reported to negatively regulate the activation of integrin αIIbβ3 by competing with talin for binding to αIIbβ3 [84]. ICln binds to the membrane-proximal KVGFFKR motif of integrin αIIb regardless of the integrin activation state, and ICln regulates platelet activation through an integrin activation-dependent subcellular redistribution mechanism [85]. Using the γ isoform of PP1c-deficient (*PP1cγ*^−/−^) mice, Gushiken et al. showed that PP1cγ mainly participates in thrombin-induced integrin αIIbβ3 inside-out signaling but not ADP or collagen-related integrin αIIbβ3 inside-out signaling. Vinculin, a marker for integrin-mediated focal adhesion complexes, inhibits Rap1-GTP-interacting adaptor molecule (RIAM) binding to talin and plays a role in inside-out signaling of αIIbβ3 [87, 94]. Using CHO cells expressing αIIbβ3, Ohmori et al. reported that vinculin induces αIIbβ3 inside-out signaling through talin-1, while it is dispensable for outside-in signaling [87]. However, conditional deletion of the vinculin gene (*Vcl*) showed that tail bleeding times in *Vcl*^*−/−*^ mice were prolonged, but platelet functions, including agonist-induced fibrinogen binding to αIIbβ3, spreading, clot retraction, platelet aggregation, and adhesion on immobilized fibrinogen or collagen, were similar to those of wild-type mice [95].

### Proteins that negatively regulate integrin αIIbβ3 activation

Several proteins are thought to bind directly to one of the integrin αIIb or β3 cytoplasmic tails to inhibit integrin αIIbβ3 activation. CIB1 plays a role in the possible negative regulation of integrin αIIbβ3 activation by binding directly to the GFFKR sequence of the αIIb cytoplasmic tail [84, 96], whereas docking protein 1 (Dok1) [71], filamin [97], and tensin 1 [98] impair integrin activation by binding directly to the β3 cytoplasmic tail [99]. There are conflicting reports on the function of CIB1 proteins in αIIbβ3 activation. Tsuboi et al. first reported that CIB1 plays an important role in the activation of αIIbβ3 in platelets [83]. When platelets were incubated with a palmitoylated peptide corresponding to the C-terminus of CIB1 (residues 179–188), no significant PAC-1 binding to αIIbβ3 was detected in the presence of physiological agonists such as ADP and thrombin. Contrasting results were reported for the overexpression of CIB1 in megakaryocytes, which completely prevented agonist-induced integrin αIIbβ3 activation, whereas overexpression of a CIB1 F173A mutant resulted in failure to interact with the αIIb cytoplasmic tail and was unable to suppress agonist-induced integrin αIIbβ3 activation. Conversely, the reduction of endogenous CIB1 via RNA interference enhanced agonist-induced integrin αIIbβ3 activation [84]. However, Denofrio et al. reported that there was no significant difference in integrin αIIbβ3 expression, agonist-induced αIIbβ3 binding to JON/A, P-selectin expression, platelet aggregation, platelet spreading, bleeding time, or FeCl_3_-induced thrombus formation between *Cib1*^+/+^ and *Cib1*^−/−^mice, possibly owing to compensation by CIB2 and CIB3 [100]. In contrast to the report of Denofrio et al., *Cib1*^*−/−*^mice showed a rebleeding phenotype and defective thrombosis due to impaired integrin αIIbβ3 outside-in signaling [101]. Dok1 is a PTB domain-containing protein. Expression of Dok1 in CHO cells expressing chimeric αIIbα6Aβ3β1A inhibited integrin activation by competing with talin for the PTB binding sites in the β1A cytoplasmic tail [102]. The integrin β3 cytoplasmic tail also has the ability to bind Dok1 [103] and impair αIIbβ3 activation. Recent studies revealed that the 14-3-3ξ/Dok1 binary complex binds to the phosphorylated cytoplasmic tail of integrin β3 and regulates integrin activation [104]. Some studies reported that knockout of Dok1 or Dok2 did not affect platelet integrin αIIbβ3 inside-out signaling, as evidenced by normal aggregation, JON/A binding, and soluble fibrinogen [105, 106]. Crystal structure studies have shown that filamin and tensin 1 can compete with talin for binding to the integrin β3 tail [107]. The roles of filamin and tensin 1 in αIIbβ3 inside-out signaling need to be further investigated using CHO cells or platelets. A gain-of-function mutation in filamin A (stop codon mutation *p. Ter2648SerextTer101*) potentiates platelet integrin αIIbβ3 activation by facilitating recruitment of talin to the β3 tail [108]. Recent studies have demonstrated that α-actinin plays a role in maintaining αIIbβ3 in an inactivated state [109]. Due to partial overlapping of α-actinin binding sites with talin binding sites in the β3 cytoplasmic tail, α-actinin association with αIIbβ3 may block the access of talin to the β3 tails [109, 110]. α-Actinin induces a kink in the transmembrane domain of integrin β3 [109–111], which maintains integrin αIIbβ3 in a low-affinity state [111].

### Agonist-induced integrin αIIbβ3 activation

**Fig. 2:**
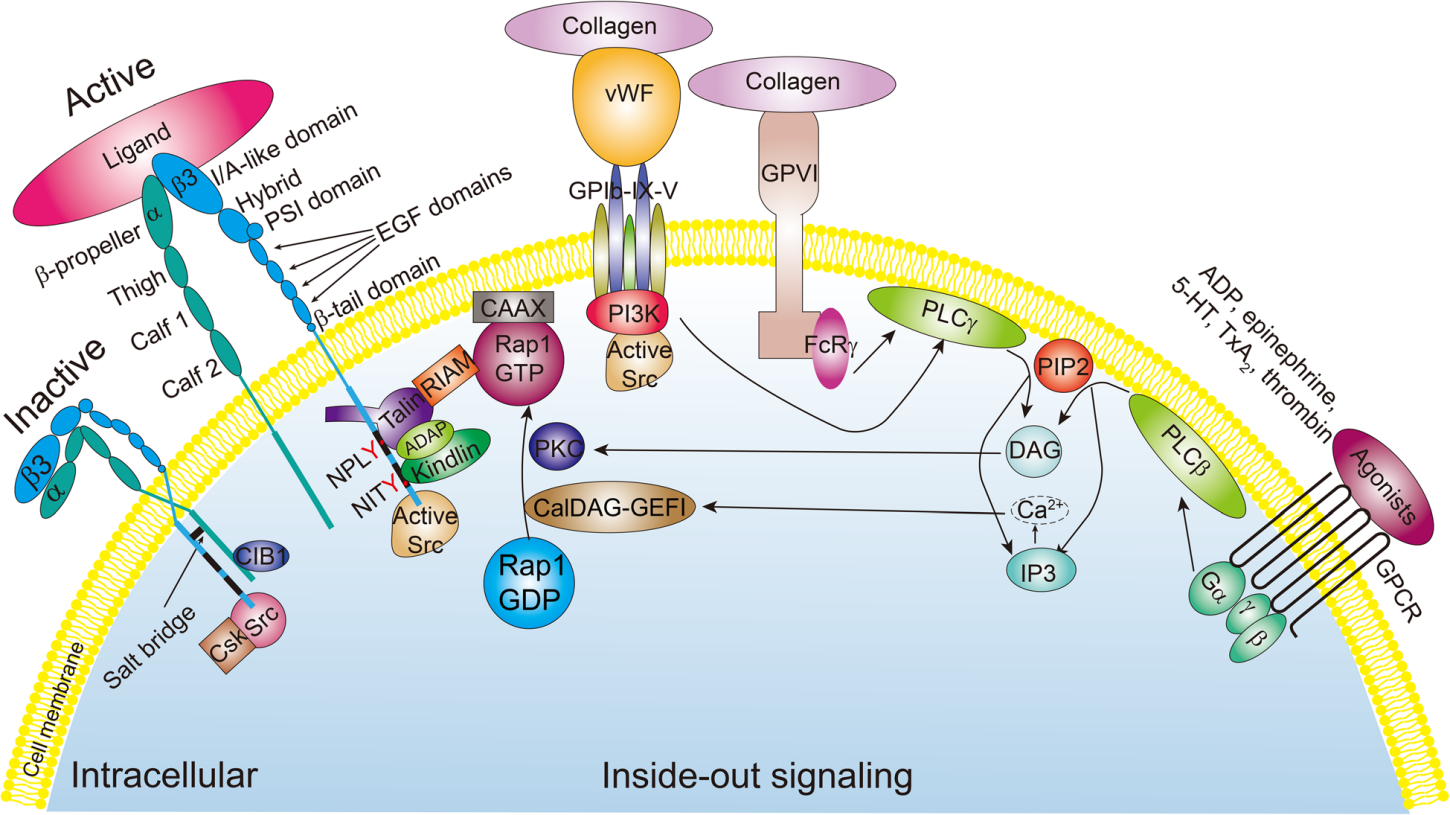
Schematic of integrin αIIbβ3 inside-out signaling in platelets. Soluble agonist (ADP, epinephrine, 5-HT, TXA_2_, and thrombin) stimulation of G protein-coupled receptors (GPCRs) triggers PLCβ activation. Collagen-bound vWF/GPIb-IX-V and collagen/GPVI interactions ultimately induce PLCγ activation. PLC hydrolyzes platelet membrane phosphatidylinositol (4,5)-bisphosphate (PI-4,5-P, PIP2) into diacylglycerol (DAG) and inositol (1,4,5)-triphosphate (IP3). IP3 induces Ca^2+^ release. DAG, together with Ca^2+^, activates CalDAG-GEFI and PKC. Activated CalDAG-GEFI along with PKC leads to the shift of Rap1-GDP to Rap1-GTP. Rap1-GTP targets the lipid membrane through farnesylation of its CAAX motif. RIAM functions as a linker between Rap1-GTP and talin, forming a Rap1/RIAM/talin complex. Complex-bound talin interacts with the integrin β3 subunit through the plasma membrane. Binding of talin-H (FERM domain) to the NPLY motif of the β3 tail disrupts the salt bridge between the αIIb and β3 subunits, leading to integrin αIIbβ3 activation, shifting from a bent to an extended conformation. Kindlin binding to the NITY motif of the β3 tail is shown. CIB1 directly binds to the αIIb cytoplasmic tail. ADAP serves as a bridging molecule between kindlin and talin, promoting platelet integrin αIIbβ3 activation

Knowledge of how agonists lead to integrin αIIbβ3 activation by talin and/or kindlin is vital for understanding inside-out signaling of αIIbβ3 (Fig. 2). The initial adhesion of platelets at the site of damaged vessel walls is mainly facilitated by GPIb-IX-V/collagen-bound vWF and/or GPVI-collagen interactions. These two interactions trigger integrin αIIbβ3 inside-out signaling and play a primary role in platelet activation. The GPIb-IX-V complex contains four type I transmembrane glycoproteins: GPIbα, GPIbβ, GPIX, and GPV. After vascular injury, circulating vWF in the plasma binds to the exposed collagen within the subendothelium through its A3 domain. The interaction of collagen and vWF-A3 enables vWF to expose the A1 domain, which is essential for collagen-bound vWF to interact with the GPIb subunit. In addition, factor XII, P-selectin, and leukocyte integrin MAC-1 are all able to bind to GPIb-IX-V and modulate integrin αIIbβ3 activation [112]. The interaction of vWF with GPIb-IX-V induces activation of the Src family kinases (Src, Lyn, and Fyn) and phosphorylation of its downstream substrates, including the Fc receptor γ-chain (FcRγ) and FcRγIIa [113–116]. PLCγ tyrosine phosphorylation is mediated by the immunoreceptor tyrosine-based activation motif (ITAM)-bearing receptors FcRγ and FcRγIIa. PLCγ is also activated by GPVI-collagen interactions through FcRγ signaling involving tyrosine kinases, such as Src and spleen tyrosine kinase (Syk) [117]. In addition to PLCγ, phosphatidylinositol-3-kinase (PI3K) is another key molecule downstream of GPVI and GPIb-IX-V [118]. Collagen- or vWF-induced signaling leads to the release of ADP, TXA2, 5-hydroxytryptamine (5-TH), and thrombin, which triggers PLCβ activation via GPCRs, such as the P2Y_1_, TP, 5-TH2A, and PAR receptors. PLCβ is downstream of GPCRs, whereas PLCγ is activated by VWF/GPIb-IX-V or collagen/GPVI interactions [118, 119]. PI3K signaling leads to Rap1 activation, which is a Ca^2+^-independent process [120]. Unlike PI3K, PLC activation hydrolyzes platelet membrane phosphatidylinositol (4,5)-bisphosphate (PI-4,5-P) into the second messengers diacylglycerol (DAG) and inositol (1,4,5)-triphosphate (IP3). In turn, IP3 releases calcium from intracellular stores through IP3 receptor (IP3-R) channels [121], increasing the Ca^2+^ concentration in the platelet cytosol. DAG and Ca^2+^ activate many isoforms of platelet protein kinase C (PKC) and Ca^2+^ diacylglycerol guanine-nucleotide-exchange factor I (CalDAG-GEFI, a guanine exchange factor for Rap1), leading to the conversion of Rap1-GDP to Rap1-GTP and the translocation of Rap1-GTP to the plasma membrane [122–124]. In *CalDAG-GEFI*^*−/−*^ mice, induction of inside-out activation of integrin αIIbβ3 by calcium ionophore, collagen, ADP, and a TXA2 analog was strongly inhibited. In contrast, thrombin-induced activation of αIIbβ3 was mildly affected [125]. This finding suggests that other molecules may transform the signal from the agonist to the αIIbβ3 cytoplasmic tails and cause αIIbβ3 activation. In addition to CalDAG-GEFI, the activation of PKC also leads to the shift of Rap1-GDP to Rap1-GTP in platelets. There are at least four PKC isoforms (α, β, δ, and θ) [126] in human platelets. Using CHO cell models, Han et al. reported that Rap1-GTP was downstream of PKCα in integrin αIIbβ3 activation [127]. Platelets from *PKCα*^*−/−*^ mice showed that PKCα was a regulator of inside-out signaling of αIIbβ3 [128] but did not play a significant role in the outside-in signaling of αIIbβ3. *Rap1b*^*−/−*^ mice demonstrated that ADP- or AYPGKF-induced integrin αIIbβ3 activation was impaired, as was FeCl_3_-dependent arterial thrombosis [129]. Interestingly, overexpression of Rap1a in CHO cells leads to αIIbβ3 activation [127], but it does not appear to be required for integrin αIIbβ3 activation in platelets [129].

Rap1 mediates inside-out activation of integrin αIIbβ3 through another effector, called Rap1-GTP-interacting adaptor molecule (RIAM), on the membrane. RIAM is a member of the Mig-10/RIAM/lamellipodin (MRL) family of adaptor molecules. RIAM recruits talin-1 to integrin αIIbβ3. Knockout of RIAM in megakaryocytes abolishes Rap1-dependent αIIbβ3 activation [130]; however, deletion of RIAM in mice does not affect αIIbβ3 activation [131]. Rap1 activation induces the formation of an “integrin activation complex” containing Rap1, RIAM, and talin, leading to αIIbβ3 activation [127, 130]. Bimolecular fluorescence complementation (BiFC) has revealed that in CHO cells, knockdown of RIAM blocks talin recruitment to αIIbβ3, whereas overexpression of Rap1a or RIAM enhances talin recruitment to αIIbβ3 [132]. RIAM acts as a scaffold that connects the membrane targeting sequences in Rap1-GTP to talin, thereby recruiting talin to the plasma membrane and activating integrins [130]. Whether kindlin is a member of the “integrin activation complex” still warrants further investigation. In addition to the Rap1/RIAM/talin complex pathway, membrane-anchored Rap1b interacts with the F0 domain of talin, triggering integrin αIIbβ3 activation in a RIAM-independent fashion [133]; however, a recent study reported conflicting results [134]. The interaction of the F0 domain of talin with Rap1b plays no evident role in talin-H-induced αIIbβ3 activation [134]. Schiemer et al. recently reported that switch region 2 of G13α had the ability to mediate talin activation from its autoinhibition station and further regulate integrin αIIbβ3 activation [135].

## Integrin αIIbβ3 outside-in signaling

**Fig. 3:**
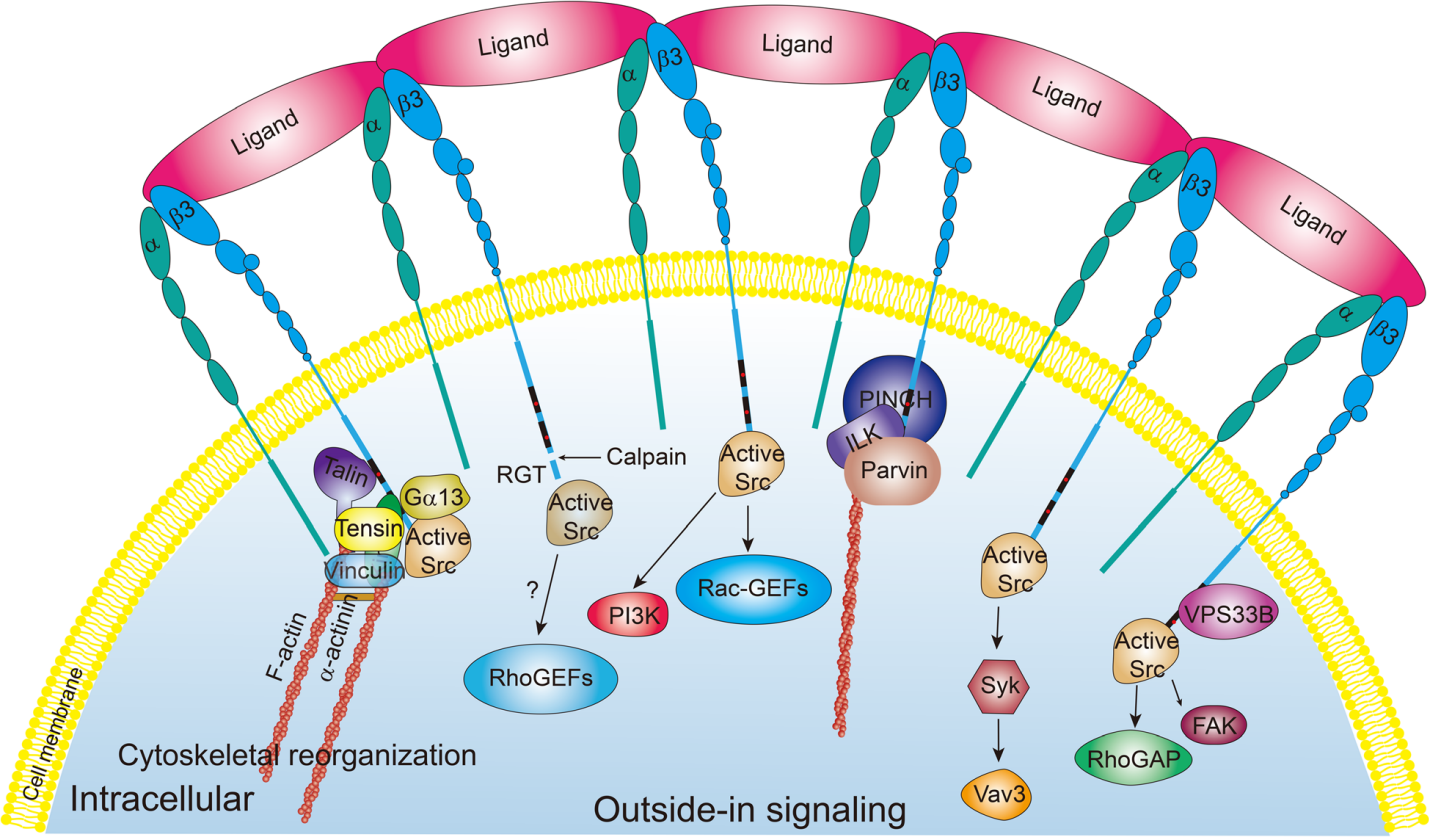
Schematic of integrin αIIbβ3 outside-in signaling in platelets. Following ligand binding to the extracellular domain of integrin αIIbβ3, integrin αIIbβ3 clustering promotes Src activation by autophosphorylation. Calpain cleaves the integrin β3 cytoplasmic tail and leads to disassociation of partly active Src from the integrin β3 tail. Src phosphorylates and supports the activation of a wide range of enzymes and signaling proteins, such as FAK, Syk kinase, RhoGAP, Rac-GEFs, RhoGEFs, and PI3K. Gα13, talin, kindlin, tensin, and vinculin provide the necessary links between the integrin β3 cytoplasmic tail and actin. Kindlin can directly couple integrin β3 to the actin cytoskeleton via the ILK/PINCH/parvin complex

**Fig. 4:**
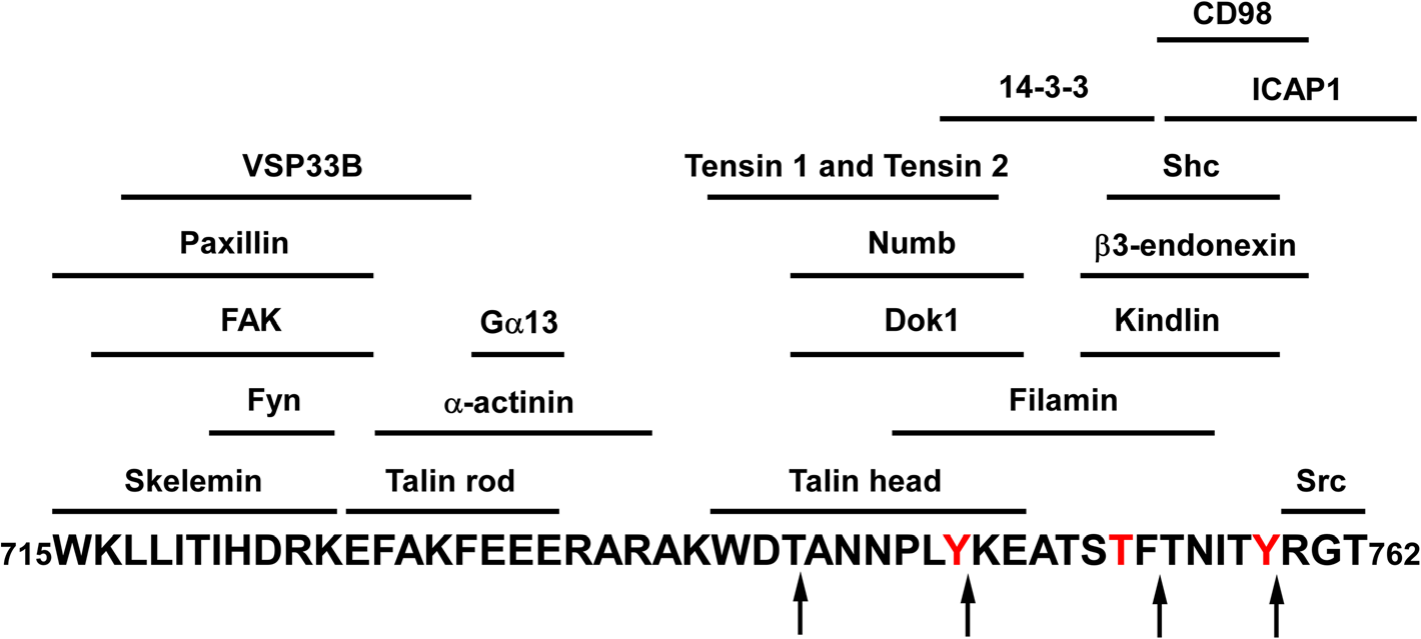
Amino acid sequence of the C-terminal tail of integrin β3, highlighting interaction sites involved in integrin αIIbβ3 bidirectional signaling. Calpain cleavage sites are indicated by arrows. Phosphorylatable amino acid residues (tyrosine and threonine) are labeled in red. Positions of the horizontal lines indicate sites on the integrin β3 cytoplasmic tail that interact with cytoplasmic signaling proteins

The outside-in signaling of integrin αIIbβ3 on platelets is triggered by the binding of soluble fibrinogen to activated integrin αIIbβ3 (Fig. 3), leading to the generation of a cascade of intracellular signaling events that mediate irreversible stable adhesion, spreading, cytoskeletal reorganization and irreversible aggregation of platelets, and subsequent thrombus growth. Similar to the inside-out signaling of αIIbβ3, outside-in signaling of αIIbβ3 requires cooperating proteins to directly or indirectly interact with the αIIbβ3 cytoplasmic tails because the cytoplasmic tails themselves lack any intrinsic enzymatic activity (Fig. 4). Many of the recent advances in our understanding of the proteins that regulate outside-in signaling of αIIbβ3 have come from mouse gene knockout studies (Table 1). To date, the identified proteins that participate in outside-in signaling of αIIbβ3 are more numerous than those involved in inside-out signaling of αIIbβ3. However, there are some proteins associated with both inside-out and outside-in signaling, such as talin and kindlin-3. The proteins that regulate outside-in signaling of αIIbβ3 can be classified into four major categories: transmembrane proteins, intracellular adaptor molecules, kinases and phosphatases, and Rho-family small GTPases.

**Table 1:** Key regulatory proteins involved in integrin αIIbβ3 bidirectional signaling

Proteins	Integrin αIIbβ3 activation	Phenotype of knockout mice	Reference
ADAP	Significantly reduced soluble fibrinogen binding	Formation of unstable thrombi, increased tail rebleeding, reduced stable attachment, and impaired cytoskeletal reorganization under shear flow	[76, 77]
CalDAG-GEFI	Impaired JON/A antibody binding	Reduced aggregation, granule secretion, and adhesive function. Mild defect in hemostasis. Impaired Rap1 activation	[104, 123–125]
c-Cbl	Null	Significantly reduced spreading on immobilized fibrinogen. Drastically impaired clot retraction	[220]
CD9	Increased soluble fibrinogen binding	Normal aggregation and α-granule release, normal hemostasis	[159]
CD63	Normal JON/A antibody binding	Normal α-granule release. Normal adhesion and thrombus formation on collagen under flow conditions	[249]
CD82	Normal JON/A antibody binding	Normal aggregation and granule secretion. Enhanced clot retraction, enhanced adhesion on fibrinogen. Reduced bleeding time and volume. Increased tyrosine phosphorylation in integrin αIIbβ3 signaling	[153]
CD84	Normal JON/A antibody binding	Normal granule secretion. Unaltered hemostatic function and arterial thrombus formation. Unaltered aggregate formation under flow. Unaltered function of *CD84*^*−/−*^ platelets in vitro	[167]
CD148	Markedly reduced JON/A antibody binding	Exhibited a bleeding tendency and defective arterial thrombosis. Markedly reduced SFK activity. Impaired spreading on fibrinogen and collagen-induced aggregate formation under flow conditions. Delayed thrombus formation	[250]
CD151	Normal soluble fibrinogen and JON/A antibody binding	Normal α-granule, dense granule secretion, and platelet adhesion. Impaired platelet aggregation and platelet spreading on fibrinogen, delayed kinetics of clot retraction, restricted cytoskeletal reorganization. Increased bleeding time and volume and rebleeding, but without spontaneous bleeding complications	[150, 156, 157]
CEACAM-1	Null	Enhanced aggregation, enhanced platelet adhesion on type I collagen but not fibrinogen, elevated granule secretion, larger and more stable thrombi	[137]
CIB1	Normal soluble fibrinogen binding	Normal aggregation and α-granule secretion, increased tail bleeding time and rebleeding, formation of unstable thrombi, impaired spreading on immobilized fibrinogen, reduced tyrosine phosphorylation of the integrin β3 tail	[93, 101]
cPLA2α	Impaired fibrinogen binding in response to CRP or the lower concentration of PAR4 peptide	Impaired collagen-induced aggregation, spreading on fibrinogen, platelet aggregation. Prolonged bleeding time	[224, 225]
Dab2	Impaired soluble fibrinogen binding	Selectively defective in thrombin-induced aggregation, platelet spreading on fibrinogen and clot retraction. Impaired ADP release. Prolonged bleeding time and impaired hemostasis and thrombosis	[179]
Dok1	Normal soluble fibrinogen and JON/A antibody binding	Normal aggregation, P-selectin surface expression. Increased clot retraction, increased PLCγ2 phosphorylation, and enhanced spreading on fibrinogen. Significantly shortened bleeding time and accelerated carotid artery thrombosis	[105]
Dok2	Normal soluble fibrinogen and JON/A antibody binding	Enhanced shear-dependent integrin adhesion in platelets. Increased platelet thrombus formation	[106]
ERp57	Impaired JON/A antibody binding	Prolonged tail bleeding time and thrombus occlusion time. Impaired platelet aggregation	[251]
ESAM	Normal JON/A antibody binding	Normal calcium mobilization, α-granule secretion and platelet spreading, more stable hemostasis. Formation of larger thrombi, increased aggregation, and more resistant to disaggregation	[145]
G6b-B	Reduced soluble fibrinogen binding	Megakaryocytes exhibited a marked reduction in spreading on fibrinogen or fibronectin, increased bleeding, failure to form normal aggregates on collagen-coated surfaces under flow condition. Impaired secretion of ATP, but not P-selectin, and reduced spreading	[140, 141]
Gα13	Normal soluble fibrinogen binding	Mutation of the Gα13-binding β3 ExE motif. Impaired stable thrombus formation. Increased tail bleeding time	[168, 169, 181]
Gas6, Gas6 receptors	Normal soluble fibrinogen binding, impaired PAC-1 binding	Failure to spread to fibrinogen, impaired dense granule secretion. No spontaneous bleeding, normal bleeding time but a tendency to repetitively rebleed. Lacked the second wave of platelet aggregation, with impaired clot retraction, reduced thrombus formation, and increased disaggregation. Reduced tyrosine phosphorylation of the integrin β_3_ tail	[160, 161, 163]
ILK	Reduced rate of soluble fibrinogen binding	Reduced α-granule secretion. Impaired aggregation, increased thrombus instability and tail bleeding time and volume	[88, 89]
JAM-A	Normal soluble fibrinogen and JON/A antibody binding	Normal α-granule secretion, enhanced thrombus formation, augmented platelet spreading and aggregation, enhanced clot retraction, shorted tail bleeding time	[147, 148]
Kindlin-3	Failed to bind soluble fibrinogen and JON/A antibody	Kindlin-3 deficiency results in severe bleeding and resistance to arterial thrombosis	[47]
Lnk	Normal soluble fibrinogen binding	Normal P-selectin expression. Reduced spreading on fibrinogen, impaired clot retraction, reduced tyrosine phosphorylation of integrin β3 tail. Impaired thrombus stability. Lnk promotes integrin αIIbβ3-mediated actin cytoskeleton reorganization	[176]
MEKK3	Impaired soluble fibrinogen binding	Reduced aggregation and granule secretion. Delayed thrombus formation and fewer microthrombi, normal tail bleeding time	[252]
Myosin	Normal soluble fibrinogen and JON/A antibody binding	Normal platelet aggregation and secretion. Increased bleeding time and absence of clot retraction. Reduced tyrosine phosphorylation of integrin β3 tail. Impaired thrombus growth, organization, and thrombus stability. Increased tail bleeding time	[172]
NLRP3	Normal JON/A antibody binding	Prolonged tail bleeding time, delayed arterial thrombus formation, impaired spreading on immobilized fibrinogen, defective clot retraction, mildly reduced platelet aggregation, normal P-selectin expression, decreased phosphorylation of Src, Syk, and PLCγ2 in response to thrombin stimulation	[13]
Palladin^+/−^	Null	Accelerated hemostasis and arterial thrombosis. Increased aggregation, spreading on immobilized fibrinogen, and rate of clot retraction	[253]
Paxillin	Enhanced JON/A antibody binding	Enhanced platelet aggregation and granule secretion, increased spreading on fibrinogen and clot retraction, increased tyrosine phosphorylation and calcium mobilization, increased thrombus formation	[79]
PDK1	Normal soluble fibrinogen binding	Diminished aggregation and spreading on immobilized fibrinogen and decreased rate of clot retraction	[254]
PECAM-1	Normal soluble fibrinogen and JON/A antibody binding	Normal α-granule secretion and aggregation, impaired spreading on immobilized fibrinogen and clot retraction, reduced tyrosine phosphorylation of FAK	[136]
PI3Kα	Null	Impaired platelet aggregation at low concentrations of CRP. Modest but significant decrease in thrombus size after superficial injury of mouse mesenteric arteries. Increased time to arterial occlusion after carotid lesion, without modification of the tail bleeding time	[219]
PKCα	Impaired soluble fibrinogen binding	Normal spreading on fibrinogen and collagen. Impaired granule release and aggregation. Markedly attenuated thrombus formation. Normal tail bleeding time	[128]
PKCβ	Normal soluble fibrinogen binding	Spread poorly on fibrinogen	[209]
PKCθ	Increased JON/A antibody binding	Partially impaired spreading on fibrinogen, but not on CRP or collagen. Increased CRP-induced granule release, unaltered platelet aggregation, and formation of significantly larger thrombi	[212, 213]
PKCι/λ	Normal JON/A antibody binding	Unaltered platelet spreading and function in vitro and in vivo under all tested conditions. Unaltered in vivo thrombus formation in *PKCι/λ*^*−/−*^ mice	[206]
PP1cγ	Moderately decreased soluble fibrinogen binding with low concentrations of thrombin or PAR4, but not ADP, collagen or CRP	Mild agonist-specific decreased aggregation. Normal granule secretion, adhesion to immobilized fibrinogen, and clot retraction. Significantly delayed thrombus formation	[86]
PTEN	Null	Shortened tail bleeding time, increased sensitivity of platelets to collagen-induced activation and aggregation	[255]
PTP-1B	Normal soluble fibrinogen binding	Poor spreading on fibrinogen and decreased clot retraction, markedly reduced thrombus formation. Prolonged tail bleeding time, but without spontaneous bleeding	[199]
Pyk2	Impaired soluble fibrinogen binding	Defective spreading on fibrinogen. Impaired aggregation and thrombus formation. Slightly prolonged tail bleeding	[221, 222]
Rac1	Null	Defective spreading on fibrinogen. Reduced thrombus formation and stability. Prolonged tail bleeding	[228, 256]
*Rap1b*	Impaired soluble fibrinogen binding	Impaired spreading on fibrinogen. Increased tail bleeding time. Reduced platelet aggregation. *Rap1b*^*−/−*^mice are protected from thrombosis in an in vivo thrombosis model	[129]
Reelin	Reduced soluble fibrinogen binding	Impaired platelet adhesion. Significantly reduced thrombus formation under high shear conditions and protected from arterial thrombosis. Normal hemostasis	[192]
RhoA	Normal JON/A antibody binding	Impaired α-granule release. Markedly prolonged tail bleeding time but also significant protection in different models of arterial thrombosis and in a model of ischemic stroke. Normal spreading on fibrinogen, impaired clot retraction, moderately reduced aggregate formation	[227]
RIAM	Normal soluble fibrinogen and JON/A antibody binding	Normal adhesion and aggregation responses under static and flow conditions. Unaltered hemostasis and arterial thrombus formation	[131]
ROCK2	Slightly impaired fibrinogen binding	Impaired adhesion and spreading on collagen, reduced aggregation. Prolonged bleeding time and delayed vascular occlusion following vessel injury	[257]
Semaphorin 4D	Normal soluble fibrinogen binding	A selective defect in collagen-induced platelet aggregation and an impaired vascular injury response. Spleen tyrosine kinase activation, and subsequent downstream events are greatly reduced in *Sema 4D*^*−/−*^ platelets. Normal spreading on collagen under flow conditions	[165]
SFKs	Normal JON/A antibody binding	Mouse platelets deficient in c-Src display impaired spreading on fibrinogen. Some redundancy with other SFKs such as Fyn and Lyn occurs, whereas Lyn is important for thrombus formation. However, Lyn also plays a negative regulatory role in cell spreading. *Fyn*^*−/−*^ platelets display delayed spreading on fibrinogen and prolonged rebleeding time. Loss of SFKs does not affect tail bleeding	[114–116, 176, 200, 214]
SHIP1	Null	SHIP1 plays a major role in regulating integrin αIIbβ3-dependent PI(3,4,5)P3 accumulation. Enhanced platelet spreading	[214]
SLP-76	Normal soluble fibrinogen binding	Impaired spreading on fibrinogen, collagen-induced platelet aggregation, and granule release. Fetal hemorrhage. Reduced tyrosine phosphorylation	[171, 182, 183]
Talin	Significantly reduced soluble fibrinogen binding	Impaired integrin αIIbβ3-mediated platelet aggregation and adhesion to collagen. Spontaneous hemorrhage and pathological bleeding	[41, 124]
TSSC6	Normal soluble fibrinogen and JON/A antibody binding	Normal platelet adhesion on fibrinogen and α-granule secretion. Increased bleeding time and volume and rebleeding. Unstable hemostasis. Impaired clot retraction, platelet aggregation, and spreading on fibrinogen	[151]
Vav1/3	Null	Impaired spreading on fibrinogen, reduced αIIbβ3-mediated PLCγ2 tyrosine phosphorylation, and reduced Ca^2+^ mobilization	[202]
Vinculin	Normal agonist-induced fibrinogen binding	Normal aggregation, adherence/spreading on immobilized fibrinogen or collagen, actin polymerization/organization, clot retraction. Prolonged tail bleeding time, but no spontaneous bleeding	[95]
VPS33B	Normal thrombin-induced soluble fibrinogen and JON/A antibody binding	Impaired spreading on fibrinogen, failure to support clot retraction. Reduced platelet aggregation and ATP secretion. Prolonged tail bleeding time	[170]
WASP	Normal fibrinogen, JON/A antibody and PAC-1 binding	Impaired adherence/spreading on immobilized fibrinogen, clot retraction and postaggregation. Primary hemostasis is normal, but rebleeding is increased	[180]

### Transmembrane proteins

#### Immunoglobulin superfamily

Platelet endothelial cell adhesion molecule-1 (PECAM-1/CD31) is a membrane-spanning immunoglobulin protein that regulates outside-in signaling, but not inside-out signaling, of integrin αIIbβ3 [136, 137]. PECAM-1 recruits SHP-1 and SHP-2 to form a signaling complex, leading to Src and FAK activation. However, exactly how Src and FAK are activated following SHP-1 and SHP-2 recruitment to PECAM-1 are unknown. PECAM-1 can also trigger the internalization of GPIb [138]. Recently, G6B and carcinoembryonic antigen-related cell adhesion molecule-1 (CEACAM-1), which bears some similarities to PECAM-1 in its cytoplasmic tail, which contains ITIM domains, and its capacity to recruit SHP-1 and SHP-2, have been shown to negatively regulate platelet thrombus formation in vitro and in vivo [137, 139–142]. Interestingly, platelets also express junctional adhesion molecule-A (JAM-A) [143] and endothelial cell-specific adhesion molecule (ESAM) [144, 145], which belongs to the cortical thymocyte marker of *Xenopus* (CTX) family of the immunoglobulin superfamily. JAM-A likely indirectly associates with integrin αIIbβ3 through CD9 [146]. In mouse knockout models, JAM-A was reported to negatively regulate αIIbβ3 outside-in signaling-mediated platelet thrombus formation through binding to Syk and inhibiting the activation of αIIbβ3-associated Src [147, 148].

#### Tetraspanin superfamily

The tetraspanins possess four conserved hydrophobic transmembrane regions: two extracellular loops and two intracellular tails (N-terminal and C-terminal). At least five members of the tetraspanin superfamily, CD151, tumor suppressing subtransferable candidate 6 (TSSC6), CD63, CD9, and CD82, have been reported to be expressed in platelets [149–153]. However, there is little information on how these tetraspanins influence αIIbβ3 outside-in signaling. So far, immunoprecipitation and Western blot studies have revealed the physical association of CD151, CD63, TSSC6, and CD9 with αIIbβ3 in platelets [150, 151, 154, 155].

Studies using murine *CD151*^*−/−*^ platelets have demonstrated that deletion of CD151 is capable of inhibiting the outside-in signaling properties of αIIbβ3, including reducing agonist-induced platelet aggregation, delaying clot retraction, diminishing platelet spreading on fibrinogen, and reducing formation of filopodia. However, *CD151*^*−/−*^ platelets display normal αIIbβ3 inside-out signaling properties, as evidenced by standard agonist-induced binding of soluble fibrinogen or JON/A antibody [150, 156, 157]. Recent studies by Orlowski et al. that used three different models for thrombus formation have confirmed that platelet CD151 is required for regulating thrombus formation in vivo [149]. CD151 forms a CD151/P2Y_12_ receptor complex and participates in integrin αIIbβ3 outside-in signaling [157]. TSSC6 regulates integrin αIIbβ3 outside-in signaling by physically associating with the P2Y_12_ receptor [158]. Early studies suggested that CD63 might inhibit integrin αIIbβ3 outside-in signaling in platelets. D545, a CD63 monoclonal antibody, modulates αIIbβ3-mediated actin cytoskeleton reorganization, inhibiting platelet spreading on immobilized fibrinogen and impairing tyrosine phosphorylation of FAK. Tyrosine phosphorylation of FAK is a downstream marker of integrin αIIbβ3 outside-in signaling. Unlike the CD151 and TSSC6 tetraspanins, CD9 does not appear to play an important role in integrin αIIbβ3 outside-in signaling but does negatively regulate integrin inside-out signaling [159]. Future studies are required to explore the role of the tetraspanins in αIIbβ3 signaling.

#### Other transmembrane proteins

Growth arrest-specific protein 6 (Gas6) is a member of the vitamin K-dependent protein family. Recent studies of *Gas6*^*−/−*^ mice have shown that Gas6 plays a role in platelet function [160–162]. *Gas6*^*−/−*^ mice have a normal bleeding time but a tendency to repetitively rebleed due to impaired αIIbβ3 outside-in signaling [160]. Interestingly, mice that have lost any one gene for the TAM family receptors (Tyro3, Axl, or Mer) display a phenotype similar to that of *Gas6*^*−/−*^ mice [163]. Once Gas6 is secreted, it binds to the TAM family receptor on the platelet surface through the C-terminal sex hormone binding globulin (SHBG)-like domain and subsequently activates downstream signaling molecules, including PI3K, Rap1, and Akt. PI3K/Akt activation leads to propagation of αIIbβ3 outside-in signaling [164]. There are some transmembrane proteins, such as Semaphorin 4D [165] and the signaling lymphocyte activation molecule (SLAM) [166, 167], that regulate integrin αIIbβ3 outside-in signaling in platelets.

### Intracellular adaptor molecules

Some intracellular adaptor molecules, such as the heterotrimeric guanine nucleotide-binding protein (G protein) Gα_13_ [168, 169], vacuolar protein sorting-associated protein 33B (VPS33B) [170], the SH2 domain-containing leukocyte protein of 76 kDa (SLP-76) [171], myosin [172], Src homology 2 domain-containing transforming protein (Shc) [173], Grb2 [174], FcγRIIa [175], lymphocyte adaptor protein (Lnk) [176], stress-activated protein kinase-interacting protein (Sin1) [177], Disabled-2 (Dab2) [178, 179], NLRP3 [13], and Wiskott-Aldrich syndrome protein (WASP) [180], are believed to be involved in integrin αIIbβ3 outside-in signaling. Gα_13_ directly binds to the integrin β3 cytoplasmic tail [168]. The spreading of CHO cells expressing αIIbβ3 on immobilized fibrinogen is inhibited by Gα_13_ siRNA interference. Gong et al. reported that platelets transfected with Gα_13_ siRNA spread poorly on immobilized fibrinogen and fail to activate Src. The myr-FEEERA peptide disrupted the Gα13/β3 interaction, thereby hampering Src activation and ultimately inhibiting αIIbβ3 outside-in signaling [181]. VPS33B, a member of the Sec1/Munc18 family, binds directly to integrin β3. Overexpression of VPS33B in CHO cells potentiated αIIbβ3 outside-in signaling but not inside-out signaling [170]. VPS33B was recently shown to function upstream of the RhoA-ROCK-MLC and Rac1-dependent pathways that lead to clot retraction and cell spreading [170]. *SLP-76*^*−/−*^ murine platelets have normal fibrinogen binding but poor spreading. In the absence of SLP-76, collagen-induced platelet aggregation and granule release, as well as the phosphotyrosine of the β3 tail, are markedly impaired [182, 183]. Myosin is known to be able to bind to the NPXY motif within β integrin cytoplasmic domains [184]. Outside-in signaling events, such as integrin β3 phosphorylation, PI-4,5-P accumulation following stimulation, and FeCl_3_-induced thrombus formation, are strongly impaired in myosin-deficient mice [172]. Fibrinogen binding to platelet αIIbβ3 induces integrin cytoplasmic domain-dependent phosphorylation of FcγRIIa, which plays an important role in αIIbβ3-mediated outside-in signaling [175]. Platelets from *Lnk*^*−/−*^mice exhibit reduced abilities in terms of full spreading on fibrinogen, fibrin clot retraction, platelet aggregation, and stable thrombus formation. Lnk is thought to mediate αIIbβ3-dependent outside-in signaling through facilitating Src phosphorylation of Fyn [176]. Shc and Grb2 are known adaptor proteins that associate with the phosphorylated β3 tails involved in outside-in signaling [173]. Disabled-2 (Dab2) is known to be expressed in megakaryocytes and platelets. Dab2 has two isoforms: p82 and p59. Ser24 of Dab2 is phosphorylated by PKCβII, PKCγ, and PKCδ, which interact with integrin β3 and ultimately inhibit integrin αIIbβ3 activation [178]. The balance between the two isoforms of Dab2 controls integrin αIIbβ3 outside-in signaling [178]. NLRP3 regulates platelet integrin αIIbβ3 outside-in signaling by decreasing thrombin-induced phosphorylation of Src/Syk/PLCγ2 [13]. Data from *WASP*^*−/−*^ mice showed that integrin αIIbβ3 outside-in signaling, such as fibrinogen and JON/A binding under agonist stimulation, is normal, whereas integrin αIIbβ3 outside-in signaling-dependent events, such as spreading on immobilized fibrinogen, fibrin clot retraction, and rebleeding, are impaired [180]. Some extracellular materials, pathogens, and other factors, such as amyloid-β [185], UV [186], *Mucor circinelloides* [187], heparin [188], and hypoxia [189], also regulate αIIbβ3 signaling. Peroxisome proliferator-activated receptor γ (PPARγ) [190], reelin [191, 192], and disulfide isomerase [193] were also reported to be involved in integrin αIIbβ3 outside-in signaling.

### Kinases, phosphatases, and Rho-family small GTPases

The maintenance of normal platelet integrin αIIbβ3 signal transduction depends on numerous kinases and phosphatases that participate in the cascade of phosphorylation and dephosphorylation. To date, more than 10 enzymes have been reported to be involved in integrin αIIbβ3 outside-in signaling. The earliest phosphorylation event after fibrinogen binding to αIIbβ3 is the activation of Src kinase. Src has been reported to directly and constitutively associate with arginine-glycine-threonine (RGT) residues of the integrin β3 cytoplasmic tail via its SH3 domain [194, 195]. In resting platelets, integrin αIIbβ3-associated Src may not be activated because tyr418 of the Src activation loop is not phosphorylated and because its SH2 domain binds to phospho-tyr529. Phosphorylation of tyr529 is maintained by C-terminal Src kinase (Csk). Interestingly, RGT-containing peptides have the ability to abrogate the interaction of Src with β3 and thus selectively inhibit integrin αIIbβ3 outside-in signaling in platelets [196, 197]. Furthermore, experimental data from the β3 (Δ760-762) knockin mouse has demonstrated that deletion of RGT residues of β3 disrupts Src-mediated αIIbβ3 signaling [198].

Following platelet activation by agonists, fibrinogen binds to αIIbβ3 and then results in tyr529 of Src being dephosphorylated by protein-tyrosine phosphatase-1B (PTP-1B) [199]. After Src activation, Syk is recruited to the β3 tail and activated by Src [200]. Some adaptor molecules, such as SLP-76, Vav1, Vav2, Val3, and SLAP-130, are downstream of Syk in αIIbβ3 outside-in signaling [171, 201, 202]. There are some controversies concerning the role of Syk in αIIbβ3 outside-in signaling. *Syk*^*−/−*^platelets adhere normally to immobilized fibrinogen [203] and fail to show appropriate aggregation responses in collagen, but thrombin-stimulated responses remain normal [204, 205].

Twelve isoforms of the PKC family are involved in most platelet functions required for thrombus formation [206]. Recent data have demonstrated that individual PKC isoforms play highly specific roles in regulating platelet functions. PKCα is an essential positive regulator of granule secretion and secretion-dependent aggregation [207, 208]. The interaction of PKCβ with αIIbβ3 is regulated by integrin occupancy, and the interaction is required for platelet αIIbβ3 outside-in signaling [209]. *PKCδ*^*−/−*^mice showed that PKCδ is a key negative regulator of filopodial formation, and a lack of PKCδ leads to enhanced platelet aggregation [210]. However, Chari et al. have reported that there is no significant difference in thrombus formation ability in the injured artery in *PKCδ*^*−/−*^ mice compared to that in wild-type mice [211]. PKCθ is constitutively associated with αIIbβ3 in human and murine platelets [160]. PKCθ is an important positive regulator in signaling between integrin αIIbβ3 and the actin cytoskeleton during platelet spreading on fibrinogen [212, 213], but not during spreading on collagen-related peptide (CRP) or collagen [213]. *PKCθ*^*−/−*^ mice have shown that PKCθ negatively regulates thrombus formation on collagen under flow [213]. However, *PKCι/λ*^*−/−*^ mice show that PKCι/λ is dispensable for αIIbβ3 bidirectional signaling [206]. Studies on murine Src homology 2 domain-containing inositol 5-phosphatase (SHIP1) knockout platelets have demonstrated that this enzyme regulates αIIbβ3-mediated platelet spreading through phosphatidylinositol (3,4,5)-triphosphate (PI (3,4,5) P3) and Src family kinases, as well as Lyn and calcium oscillation [214]. PI (3,4,5) P3 binds to Rasa3 and reduces Rasa3 Rap1GAP activity, thus facilitating CalDAG-GEFI-mediated Rap1 activation and regulation of αIIbβ3 outside-in signaling [215]. The activation of PI3K and internal calcium pathways are thought to be crucial for αIIbβ3 outside-in signaling [216]. *PI3Kγ*^*−/−*^ platelets have demonstrated a diminished ability to reorganize the cytoskeleton, spread on fibrinogen, and form stable thrombi in vivo using a FeCl_3_-induced carotid injury model [217, 218]. The absence of PI3Kα leads to a reduction in thrombus size and increased arterial occlusion time but does not alter the tail bleeding time [219]. The E3 protein-ubiquitin ligase c-Cbl associates with the class I PI3K p85 regulatory subunit, regulating αIIbβ3 integrin outside-in signaling through Src family kinase (SFKs), Syk, and Pyk2 [19]. *Pyk2*^*−/−*^ platelets show a significant defect in integrin αIIbβ3 outside-in signaling, similar to the loss of c-Cbl or PI3Kβ activity [220–223]. Group IVA cytosolic phospholipase A_2_ (cPLA_2_α) and vimentin, a cPLA_2_α binding partner, are constitutively associated with αIIbβ3 in mouse and human platelets [224]. The data from the cPLA_2_α^−/−^platelets demonstrated that αIIbβ3 outside-in signaling was impaired and inside-out signaling was partially impaired [224, 225]. Khatlani et al. recently reported that the interaction of the catalytic subunit of protein phosphatase 2A (PP2Ac) with the adaptor protein Cbl-interacting protein of 85 kDa (CIN85) supports integrin αIIbβ3 outside-in signaling by suppressing phosphatase activity [226]. The Rho-family GTPases RhoA [227], Ras-related C3 botulinum toxin substrate 1 (Rac1) [228], and cell division control protein 42 (Cdc42) [229] are important for integrin-mediated platelet shape changes, but their precise role in αIIbβ3 outside-in signaling has been controversial [19].

## Therapeutic agents targeting integrin αIIbβ3 in clinical use

Therapeutic agents targeting integrin αIIbβ3, both approved for clinical use and under development, are shown in Table 2. Currently, three therapeutic agents, consisting of integrin αIIbβ3 antagonists, the antibody fragment abciximab, and two small molecule inhibitors (eptifibatide and tirofiban), have been approved for clinical use in most countries. Abciximab (Reopro) is the Fab fragment of the mouse/human chimeric monoclonal antibody 7E3 that binds to an epitope near the ligand binding site of integrin β3. The steric hindrance resulting from the binding of abciximab to integrin αIIbβ3 prevents the interaction of fibrinogen and other ligands with integrin αIIbβ3, interfering with platelet aggregation and thrombosis. Abciximab has a nearly equal affinity for blocking either integrin αIIbβ3 or αvβ3 [230]. In addition, abciximab also reacts with a member of the β2 integrin subfamily of leukocyte integrins, called Mac-1 (CD11b/CD18, αMβ2) [231]. This feature gives abciximab anti-inflammatory and antiproliferative properties, but the clinical implications are unclear. Eptifibatide (Integrilin) is an 832 Da cyclic heptapeptide containing a lysine-glycine-aspartic acid (KGD) sequence, based on the structure of snake venom barbourin [232]. Tirofiban (Aggrastat) is a 495 Da synthetic compound (an l-tyrosine derivative) that acts as an RGD mimetic. The EPIC trial showed a reduced frequency of restenosis in high-risk angioplasty patients who received abciximab infusion [233]. Three phase 3 clinical trials (EPIC, EPILOG, and CAPTURE) showed that abciximab is effective in the prevention of ischemic cardiac complications, either in patients undergoing percutaneous coronary intervention or in patients with unstable angina (UA)/non-ST-elevation myocardial infarction (NSTMI) that was unresponsive to conventional therapy [234]. In recent years, tirofiban and eptifibatide have been introduced in clinical practice. Eptifibatide and tirofiban have also been approved for use in unstable angina, as well as angioplasty. The STRATEGY, MULTI-STRATEGY, and EVA-AMI trials demonstrated similar clinical outcomes between eptifibatide and abciximab in patients undergoing primary angioplasty [230]. Eptifibatide and tirofiban were developed to be used in patients with acute coronary syndrome (ACS) as a bridging therapy to revascularization. Eptifibatide and tirofiban were used directly in the catheterization laboratory immediately prior to PCI [235]. All three integrin αIIbβ3 antagonists are administered intravenously, but several oral active agents have been extensively investigated. Orbofiban, sibrafiban, xemilofiban, lefradafiban, and roxifiban are all experienced on phase II or phase III clinical trials. However, these oral αIIbβ3 antagonists are associated with a prolonged bleeding time, an increase in the incidence of thrombocytopenia, and a 30–35% increase in mortality, including cardiovascular mortality, potentially outweighing the beneficial effects [236]. Orally active antagonists have not yet been approved due to these adverse effects, as well as the fact that oral antagonists have exhibited no significant advantage compared to aspirin in large-scale clinical trials (totaling 33,326 subjects) [237]. For a more in-depth examination of integrin αIIbβ3 antagonists, several comprehensive reviews have been selected for further reading [238–240]. In addition to integrin αIIbβ3 antagonists, some potential therapeutic agents (cilengitide, MRL-123) targeting the integrin αvβ3 molecule have been extensively investigated for anti-cancer or osteoporosis [238].

**Table 2:** Therapeutic agents targeting the integrin αIIbβ3 molecule in clinical use and preclinical studies

Class	Agent	Synonyms	Status	References
Monoclonal antibody	Abciximab	ReoPro, Clotinab, CentoRx	Approved	[230]
	YM337	Null	No development reported	[258]
KGD sequence	Eptifibatide	Intrifiban, SB-1, Sch-60936, Integrilin	Approved	[232]
RGD sequence	MK-0852	L-367073	No development reported	[259]
	G4120	Null	No development reported	[260]
	DMP-728	Null	No development reported	[261]
Nonpeptide inhibitors	Tirofiban	L-700462, MK-383, Aggrastat	Approved	[262]
	Lamifiban	Ro-449883	Not approved	[263]
	GR144053	Null	No development reported	[264]
Oral agents	Xemilofiban	SC-54684; SC-54701 is the active component of xemilofiban	Not approved	[265]
	Orbofiban	SC-57099B, CS-511; SC-57101 is the active component of orbofiban	Not approved	[266]
	Sibrafiban	Null	Not approved	[267]
	Lotrafiban	Null	Not approved	[268]
	Lefradafiban	BiBu-104; fradafiban is the active component of lefradafiban	Not approved	[238]
	Roxifiban	DMP754	Not approved	[269]
	Cromafiban	CT-50352	Not approved	[238]
	FK-633	Null	Not approved	[238]
	Elarofiban	RWJ-53308	Not approved	[238, 270]
	SR-121787	Null	Not approved	[238, 271]
	Alnidofibatide	PRP-109891, Klerval	Not approved	[272]
Others	ANTP266	Null	Preclinical studies	[246]
	RUC-1, RUC-2	Null	Preclinical studies	[273, 274]
	PLT/uPA-T	Null	Preclinical studies	[244]
	scFvSCE5-scuPA	Null	Preclinical studies	[243]
	Targ-CD39	Null	Preclinical studies	[275]
	myr-FEEERA	Null	Preclinical studies	[181]
	RGT-containing peptides	Null	Preclinical studies	[196, 197]

## Innovative agents/concepts targeting integrin αIIbβ3 and its signaling pathways

Because of the marked inhibition of platelet function, integrin αIIbβ3 antagonists can increase bleeding risk, although many studies suggest that these antagonists do not significantly increase the risk of life-threatening bleeding when compared to standard unfractionated heparin [235]. Severe thrombocytopenia is associated with all three currently approved integrin αIIbβ3 antagonists [241]. Thus, integrin αIIbβ3 antagonists must act in a narrow therapeutic window to prevent uncontrolled bleeding. The integrin αIIbβ3 antagonists currently in clinical use have been reported to cause conformational changes of αIIbβ3, inducing fibrinogen binding (priming) and eliciting outside-in signaling, thereby causing paradoxical platelet activation [242]. Currently, three novel and attractive concepts for avoiding bleeding risk are under development. (1) The single-chain variable fragment (scFv) of anti-integrin αIIbβ3 fused to an anticoagulant, fibrinolytic drugs, and CD39 is being developed. In preclinical studies, the prodrugs PLT/uPA-T and scFvSCE5-scuPA effectively inhibited thrombosis without affecting hemostasis [243, 244]. Targ-CD39 (CD39 recombinantly fused to an activated αIIbβ3-specific scFv) also demonstrates strong antithrombotic potency without hemostatic disturbance [245]. (2) Small molecules, such as RUC-1 and RUC-2, which selectively inhibit αIIbβ3 binding to fibrinogen to avoid a conformational change of the integrin αIIbβ3, are also being developed. Unlike classic agents, RUC-1 and RUC-2 bind to the metal ion binding site of β3 to inhibit fibrinogen binding. RUC-1 and RUC-2 do not induce a conformational change of integrin β3. As a result, they do not “prime” αIIbβ3 to bind its ligands. These small molecules that selectively inhibit fibrinogen binding to integrin αIIbβ3 have shown potent antithrombotic effects with low bleeding risk [246, 247]. (3) Targeting the integrin αIIbβ3 outside-in signaling pathways instead of the integrin αIIbβ3 molecule itself is another approach. Transgenic animals with impaired integrin αIIbβ3 outside-in signaling displayed a similar phenotype of reduced thrombosis potential, without excessive bleeding [248]. Thus, blocking integrin αIIbβ3 outside-in signaling has a potential advantage for the design of new antithrombotic therapies. A major advantage of targeting integrin αIIbβ3 outside-in signaling may be unaffected primary platelet adhesion and the first wave of reversible aggregation, which is critical for hemostasis but can reduce the size of a thrombus to prevent vessel occlusion [181, 196]. A recent study showed that the myr-FEEERA peptide selectively inhibits the Gα13-integrin β3 interaction, ultimately impairing Src activation and thereby inhibiting integrin αIIbβ3 outside-in signaling [181]. Both eptifibatide and the myr-FEEERA peptide inhibit laser-induced arteriolar thrombosis and FeCl_3_-induced occlusive carotid artery thrombosis. Eptifibatide also dramatically prolongs tail bleeding and increases blood loss; however, the myr-FEEERA peptide had no such adverse side effects [181]. Our studies have demonstrated that RGT-containing peptides have the ability to selectively inhibit integrin αIIbβ3 outside-in signaling through physical dissociation of the Src/β3 interaction in platelets [196, 197]. The results from ex vivo flow-based assays show that RGT-containing peptides inhibit thrombus formation under high shear rates but not under intermediate or low shear rates. The RGT peptide, its derivatives, and its analogs may have the potential to be developed into novel antithrombotic agents that specifically disrupt integrin αIIbβ3 outside-in signaling. However, it is still important to consider and investigate potential off-target effects caused by selective targeting of the Gα13-β3 and Src-β3 interactions.

## Conclusions

The development of proteomics, biophysics, and gene knockout/knockin technologies has uncovered an increasing number of proteins that participate in the bidirectional signaling of integrin αIIbβ3 and has begun to shed light on their mechanisms and roles in regulating integrin αIIbβ3 signaling. Given the importance of integrin αIIbβ3 bidirectional signaling in maintaining proper platelet function, examining the complex regulatory relationship between these interacting proteins can prove immensely important for understanding the mechanisms of platelet activity, as well as for developing new therapies for cancer and thrombosis based on a deeper knowledge of the underlying physiology. Until now, the complex stoichiometric and spatiotemporal dynamics between integrin αIIbβ3 and its regulatory proteins have remained obscure, but promising new techniques have already presented new opportunities to learn more. Considerable efforts are still needed to fully explore how integrin αIIbβ3 interacts with its regulatory proteins, how its regulatory proteins interact with one another in space and time, and how therapeutic agents targeting integrin αIIbβ3 and its pathways can provide therapeutic benefits while minimizing adverse side effects.

## Abbreviations

5-TH: 5-Hydroxytryptamine
ACS: Acute coronary syndrome
ADAP: A hematopoietic-specific adapter protein
ADP: Adenosine diphosphate
BiFC: Bimolecular fluorescence complementation
CalDAG-GEFI: Ca^2+^ diacylglycerol guanine-nucleotide-exchange factor I
Cdc42: Cell division control protein 42
CEACAM-1: Carcinoembryonic antigen-related cell adhesion molecule-1
CHO: Chinese hamster ovary
CIB1: Calcium- and integrin-binding protein 1
CIN85: Cbl-interacting protein of 85 kDa
cPLA_2_α: Group IVA cytosolic phospholipase A_2_
CRP: Collagen-related peptide
Csk: C-terminal Src kinase
CTX: Cortical thymocyte marker of *Xenopus*
Dab2: Disabled-2
DAG: Diacylglycerol
Dok: Docking protein
EPIC: European Prevalence of Infection in Intensive Care
ESAM: Endothelial cell specific adhesion molecule
FAK: Focal adhesion kinase
FcRγ: Fc receptor γ-chain
FERM: 4.1, Ezrin, radixin, moesin
Gas6: Growth arrest specific protein 6
GP: Glycoprotein
GPCR: G protein-coupled seven-transmembrane domain receptor
Grb2: Growth factor receptor bound protein 2
GT: Glanzmann’s thrombasthenia
HPC: Hematopoietic progenitor cell
HSC: Hematopoietic stem cell
IBS: Integrin-binding site
ICln: Chloride channel regulatory protein
ILK: Integrin-linked kinase
IP3: Inositol (1,4,5)-triphosphate
IP3-R: P3 receptor
IPP: ILK/PINCH/parvin
ITAM: Immunoreceptor tyrosine-based activation motif
ITIM: Immunoreceptor tyrosine-based inhibitory motif
JAM-A: Junctional adhesion molecule-A
KGD: Lysine-glycine-aspartic acid
Lnk: Lymphocyte adaptor protein
mAb: Monoclonal antibody
MEKK3: Mitogen-activated protein kinase/extracellular-regulated kinase kinase kinase-3
MRL: Mig-10/RIAM/lamellipodin
NLRP3: NACHT, LRR, and PYD domain-containing protein 3
NSTMI: Non-ST-elevation myocardial infarction
PCI: Percutaneous coronary intervention
PDK1: 3-Phosphoinositide-dependent protein kinase-1
PECAM-1: Platelet endothelial cell adhesion molecule-1
PI3K: Phosphatidylinositol-3-kinase
PI-4,5-P: Phosphatidylinositol (4,5)-bisphosphate
PIPK1γ: Phosphatidylinositol 4-phosphate 5-kinase isoform 1γ
PKC: Protein kinase C
PMA: Phorbol 12-myristate 13-acetate
PP1cγ: Catalytic subunit of protein phosphatase 1 γ
PP2Ac: Catalytic subunit of protein phosphatase 2A
PPARγ: Peroxisome proliferator-activated receptor γ
PTB: Phosphotyrosine-binding domain
PTEN: Phosphatase and tensin homolog deleted on chromosome ten
PTP-1B: Protein-tyrosine phosphatase-1B
PXN: Paxillin
Pyk2: Proline-rich tyrosine kinase 2
Rac1: Ras-related C3 botulinum toxin substrate 1
RGT: Arginine-glycine-threonine
RhoA: Rho-family GTPase
RIAM: Rap1-GTP-interacting adaptor molecule
SFK: Src family kinase
SHBG: Sex hormone binding globulin
Shc: Src homology 2 domain-containing transforming protein
SHIP: Src homology 2 domain-containing inositol 5-phosphatase
Sin1: Stress-activated protein kinase-interacting protein
SLAM: Signaling lymphocyte activation molecule
SLP-76: SH2 domain-containing leukocyte protein of 76 kDa
Syk: Spleen tyrosine kinase
TM: Transmembrane
TSSC6: Tumor suppressing subtransferable candidate 6
TXA2: Thromboxane A2
UA: Unstable angina
UV: Ultraviolet
VEGF: Vascular endothelial growth factor
VPS33B: Vacuolar protein sorting-associated protein 33b
vWF: von Willebrand factor
WASP: Wiskott-Aldrich syndrome protein
